# A Comprehensive Analysis of the Thrombin Binding Aptamer Containing Functionalized Pyrrolo-2’-deoxycytidines

**DOI:** 10.3390/ph14121326

**Published:** 2021-12-18

**Authors:** Weronika Kotkowiak, Zofia Jahnz-Wechmann, Anna Pasternak

**Affiliations:** Department of Nucleic Acids Bioengineering, Institute of Bioorganic Chemistry, Polish Academy of Sciences, Noskowskiego 12/14, 61-704 Poznan, Poland; kawecka@ibch.poznan.pl (W.K.); jahnz@ibch.poznan.pl (Z.J.-W.)

**Keywords:** G-quadruplex, thrombin binding aptamer, thermodynamics, circular dichroism, anticoagulant activity, anticancer agents

## Abstract

Aptamers constitute an answer for the growing need for targeted therapy development. One of the most well-known representatives of this group of compounds is thrombin binding aptamers (TBA) targeted towards thrombin. The TBA inhibitory activity is determined by its spatial arrangement, which consists of two G-tetrads linked by two shorter TT loops and one longer TGT loop and folds into a unimolecular, antiparallel G-quadruplex structure. Interesting properties of the aptamer can be further improved via the introduction of a number of chemical modifications. Herein, a comprehensive analysis of the influence of pyrrolo-2’-deoxycytidine (Py-dC) and its derivatives on TBA physicochemical and biological properties has been presented. The studies have shown that the presence of modified residues at the T7 position of the TGT loop has only minor effects on TBA thermodynamic stability without affecting its folding topology. All analyzed oligomers exhibit anticoagulant properties, but only aptamer modified with a decyl derivative of Py-dC was able to inhibit thrombin activity more efficiently than unmodified, parental compounds. Importantly, the same compound also possessed the potential to effectively restrain HeLa cell line growth.

## 1. Introduction

The latest trends in modern medicine rely on the recognition of a molecular basis of disease and the development of drugs based on this knowledge. The challenges can be met by the design and synthesis of compounds capable of exerting a therapeutic effect based on an in-depth understanding of the structure and mechanism of action of the target molecule. The group of compounds, which perfectly enroll in the above trends, are aptamers. They constitute a group of single-stranded oligonucleotides, whose sequence determines folding into a peculiar tertiary structure [1]. The specific shape of aptamers ensures the high specificity and selectivity of interactions with target molecules [2,3,4]. Due to their high biological activity, ease of chemical synthesis, and advantageous dissociation constant, aptamers have found a number of potential applications in medicine, with the superior ability to be highly specific drugs characterized by reversibility of action and extended half-life.

**Figure 1:**
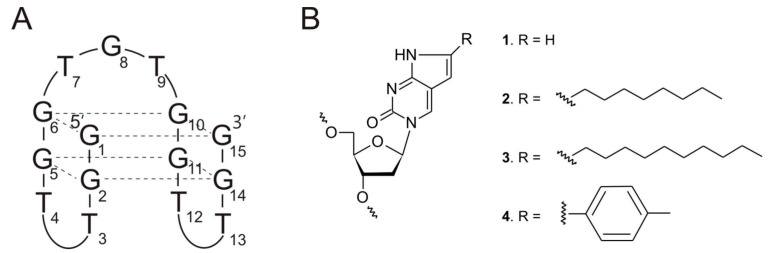
Schematic representation of TBA and modified nucleoside residues. Structure of thrombin binding aptamer, TBA (**A**) and pyrrolo-2’-deoxycytidine (Py-dC) and its derivatives (**B**).

One of the first and the most well-known representatives of aptamers is the thrombin binding aptamer (TBA), aimed at inhibiting thrombin activity [5]. The TBA adopts the structure of an antiparallel, intramolecular G-quadruplex with a chair-like conformation and a core composed of two G-tetrads stabilized by potassium ions, connected by two TT edge-wise loops and one TGT loop (Figure 1A) [6,7]. The target protein not only plays a crucial role in processes occurring in physiological conditions but also takes part in the pathogenesis of numerous diseases, such as atherosclerosis [8], cancer [9], and lung fibrosis [10], which makes it an excellent therapeutic target. One of the most important features of thrombin, in the context of TBA action, is its structure. The protein is composed of four basic structural domains: the proteolytic site (active site), the sodium ion binding site, and two positively charged external sites: fibrinogen-binding site (exosite I) and heparin-binding site (exosite II) [11,12]. Comprehensive structural studies showed that TBA binds to thrombin exosite I via its two TT-loops, which act as a pincer-like motif by grabbing the protruding fragment of the fibrinogen recognition site region [13,14]. By the last decade, a great number of chemical modifications were introduced into TBA in order to improve its biological properties [15,16,17,18,19,20,21,22,23,24,25,26,27].

The pyrrolo-2’-deoxycytidine (Py-dC) and its derivatives are a group of compounds, which exhibit fluorescent properties. This feature allows for the analysis of DNA and RNA structures and dynamics [28]. The compounds are characterized by the presence of an additional five-membered pyrrole ring attached to cytidine residue (Figure 1B, 1). What is more, the derivatives can be further extended by adding additional substituents: aliphatic, linear side-chains (Figure 1B, 2–3), or phenyl groups (Figure 1B, 4). It was recently proven that the introduction of the modified nucleotide residues into hairpin stems could facilitate distinction between single- and double-stranded fragments of DNA, with only minor perturbation in its thermodynamic stability [29].

Herein, the introduction of pyrrolo-2’-deoxycytidine (Py-dC) and its derivatives into the TBA loop has been presented for the first time. In the further course of the research, the influence of single substitution with modified nucleotide residues at the TBA T7 position on thermodynamic and biological properties of the aptamer has been evaluated. Importantly, the alteration of the TBA T7 position was previously reported to have a particularly beneficial influence on the aptamer biological activity, and therefore this localization was selected to be modified by Py-dC and its derivatives [30,31]. In detail, the potential variation in the TBA folding topology and changes in G-quadruplex thermodynamic stability induced by the presence of Py-dC and its derivatives has been analyzed. What is more, the determination of the anticoagulant and anticancer potential of the new, modified TBA variants has also been performed.

## 2. Results and Discussion

### 2.1. Thermodynamic Properties of TBA Variants

Thermodynamic studies performed for all new variants of TBA revealed only minor changes in the stability of G-quadruplex. In general, the presence of bicyclic 2′-deoxycytidine and its derivatives at position T7 of the TBA is slightly less energetically favorable in reference to the unmodified aptamer, causing an increase in Gibbs free energy values (Table 1). It was previously reported that pyrrolo-2′-deoxycytidine (Py-dC) residue slightly stabilizes hairpin structures only when placed within the stem fragment as a consequence of improved stacking interactions [29]. The presence of the large surface of the aromatic ring of Py-dC residue at the T7 position of the TBA causes minor destabilization of the G-quadruplex (ON1, ΔΔG°_37_ = 0.25 kcal/mol). The functionalization of Py-dC with aliphatic, linear side-chains does not significantly influence the thermodynamic effect observed for Py-dC. However, a slight reduction in the unfavorable outcome caused by Py-dC can be observed (ΔΔG°_37_ = 0.17 and 0.23 kcal/mol for octyl (ON2) and decyl (ON3) derivative of Py-dC, respectively). In general, the detailed thermodynamic analysis of other TBA analogues suggests that the type of natural heterocyclic base, i.e., thymine, cytosine, adenine, and guanine, placed at position T7, seems not to be crucial for thermodynamics of the TBA G-quadruplex [31]. Therefore, the destabilization effect caused by modified residues described herein is rather attributable to a steric hindrance caused by more bulky heterocyclic moieties in reference to thymine, which is present in regular TBA. Additionally, according to Borbone et al., thymidine residues within a TGT loop of unmodified TBA prefer anti-conformation of the N-glycosidic bond with carbonyl oxygen flipped out of the sugar surface [32]. On the contrary, bicyclic derivatives similar to Py-dC were shown to possess opposite conformation of the N-glycosidic bond with the carbonyl oxygen facing above the sugar [33]. Thus, the destabilization of TBA set off by the presence of Py-dC, and aliphatic derivatives might also be due to conformational change within nucleoside residue present at position T7. Importantly, the most significant destabilization occurs in the presence of the phenyl derivative of Py-dC within the TBA loop (ON4), inducing a decrease in TBA thermodynamic stability by 0.57 kcal/mol. An analogous thermodynamic profile was described previously for the hairpin structures modified with phenyl Py-dC within a stem and loop fragment [29]. Moreover, it was reported that thymidine residue at position T7 of the TBA structure is pointed towards a solution and, as a consequence, it is not directly involved in any interactions with the remaining residues within the G-quadruplex structure [34]. Thus, the increased destabilization caused by the phenyl derivative in reference to Py-dC at position T7 might be driven by larger steric hindrance induced by the presence of the bulky phenyl group, which was similarly observed for the phenyl Py-dC modified hairpin loop [29]. Furthermore, the analysis of enthalpy–entropy compensation indicates that the presence of modified residues at position T7 causes unfavorable enthalpy changes that overcome favorable entropic effect, resulting in overall destabilization of the TBA. Since the Py-dC derivatives show increased hydrophobic character in comparison to regular thymidine, the favorable entropic effect can be attributable in part to the disruption and release of ordered water molecules within the G-quadruplex structure.

### 2.2. The Analysis of TBA Variants Folding Topology

The attempts to correlate the thermodynamic stability of novel TBA variants with possible changes to the G-quadruplex structure induced by the presence of modified nucleotide residues were made in this work. As mentioned above, TBA forms an antiparallel, intramolecular G-quadruplex with a chair-like conformation, and such a strictly defined structure seems to be substantial for the interactions of the aptamer with the thrombin molecule. The effect of bicyclic nucleotide derivatives on folding topology of analyzed TBA variants were determined via circular dichroism (CD) spectra analysis. This useful and widely applied technique allow distinguishing between different G-quadruplex folding topologies based on various CD spectra patterns. The parallel and antiparallel structures are characterized by distinct CD profiles [35]. The parallel G-quadruplexes have a positive ellipticity near 260 nm and a negative ellipticity near 240 nm, whereas antiparallel G-quadruplexes have a maximum near 240 nm and 295 nm, and a minimum around 265 nm.

**Figure 2:**
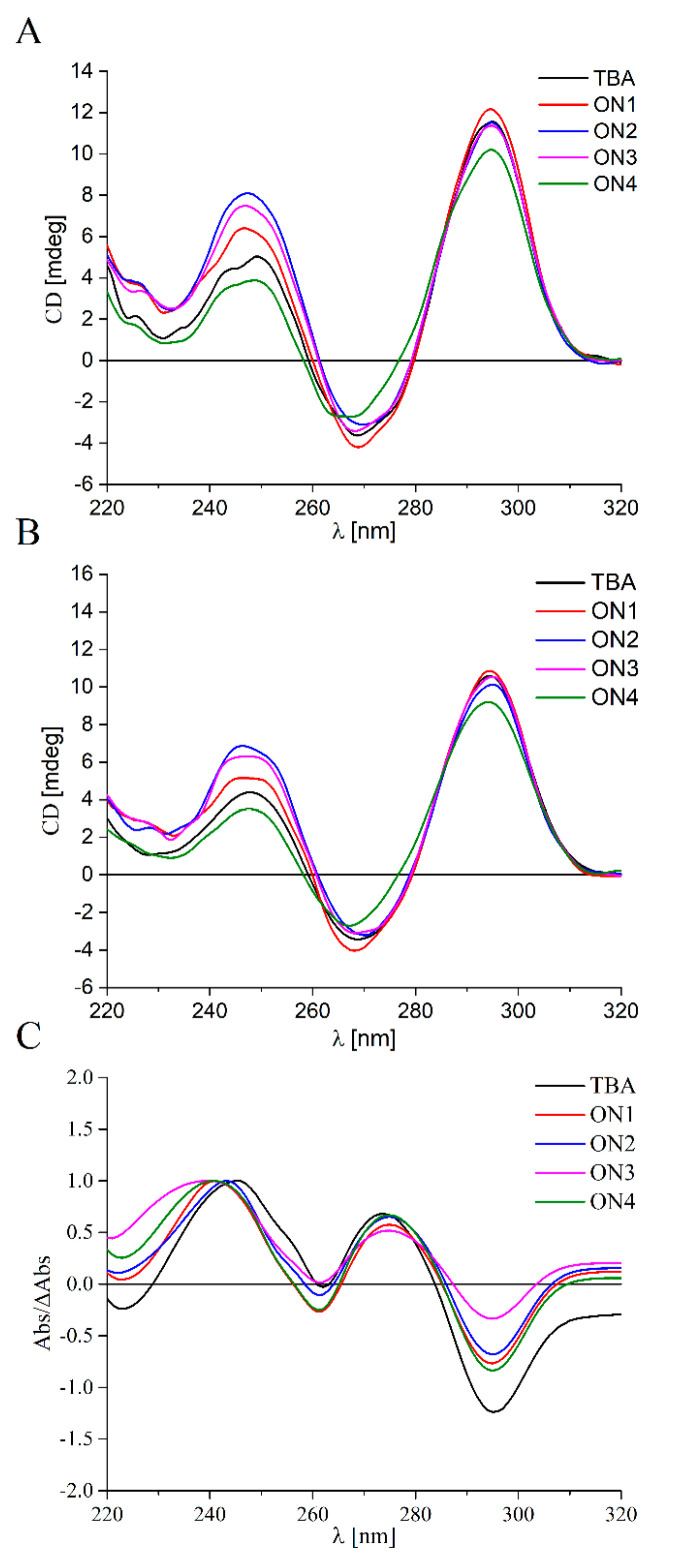
Circular dichroism spectra performed at 4 °C (**A**) and 37 °C (**B**) and thermal difference spectra (**C**) of unmodified TBA (black lines) and TBA variants modified with bicyclic Py-dC (ON1, red line), octyl Py-dC (ON2, blue line), decyl Py-dC (ON3, magenta line) and phenyl Py-dC (ON4, green line).

The CD analysis for all analyzed oligomers was conducted at two temperatures, 4 °C and 37 °C. The shape of the spectra at 4 °C for all TBA variants was characterized by the presence of two maxima near 245 and 295 nm and one minimum around 270 nm (Figure 2A). This specific pattern of the CD spectra indicates that the oligomers possess an antiparallel G-quadruplex folding topology. What is more, the CD analysis performed at 37 °C reveals that this structure was preserved in a physiological temperature. The spectra had a similar shape to those conducted at 4 °C, i.e., two positive peaks around 245 and 295 nm and one negative peak around 270 nm (Figure 2B). Based on the above results, and taking into consideration previously published data [16,30,31], it can be concluded that the introduction of modified nucleotide residues does not change the TBA folding topology. What is more, it can be assumed that the antiparallel G-quadruplex structure is preserved at a physiological temperature, which gives a great opportunity for the application of the analyzed oligomers for medical purposes.

In order to verify results obtained from the analysis of the CD spectra, thermal difference spectra (TDS) were applied [36]. The overall patterns of TDS are for a particular RNA or DNA structure and reflect the subtleness of stacking interactions, which occurs uniquely for a particular type of nucleic acid structure. TDS are often used as a supplement to the CD spectra technique due to the fact of being a convenient, reproducible, and informative method to analyze G-quadruplex folding with ease.

The shape of the resultant TDS spectra of the oligonucleotides was characterized by the presence of two positive bands around 245 and 275 nm and two negative bands around 260 and 295 nm (Figure 2C). The profile of TDS was in accordance with previously published data for unmodified TBA [16,30,31] and proved that analyzed TBA variants possess an antiparallel G-quadruplex structure. The TDS results were in line with those derived from the CD analysis and thermodynamic studies and evidenced that the introduction of modified derivatives of Py-dC had no influence on the overall G-quadruplex folding topology.

### 2.3. Anticoagulant Properties of Modified Thrombin Binding Aptamer Variants

Coagulation is a physiological process guided by thrombin, whose essence is the conversion of fibrinogen into fibrin and subsequently a fibrin clot formation. The efficiency of this process can be assessed by the determination of thrombin time (TT). This parameter is equal to the amount of time, which passed from the addition of the thrombin to plasma till the clot formation. It is a useful approach to estimate not only the normal value of human clotting time but also the effectiveness of anticoagulant action. In the latter case, the thrombin time is extended and corresponds to the better anticoagulant properties [16,31,37].

Herein, the designation of thrombin time was used to analyze the influence of modified nucleotide residues on anticoagulant properties of TBA variants. The measurements were conducted in one concentration for each variant (final concentration was 0.165 µM), and the results were expressed in a relative parameter named anticoagulant effect (AE), calculated by subtraction of TT value for control plasma without aptamers from TT value for plasma with aptamers. The data analysis revealed that all analyzed variants exhibited anticoagulant properties. The most significant prolongation of TT, also in comparison to unmodified TBA, was observed in the variant modified with a decyl derivative of Py-dC containing a long aliphatic, linear side-chain (Table 2, the AE for ON3 and TBA was equal to 30 s and 26.2, respectively). The reduction of the length of the aliphatic, linear side-chain to eight carbon atoms caused a decrease in the TBA variant anticoagulant properties and the AE was equal to 20 s (Table 2, ON 2). Noteworthy, the presence of bicyclic pyrrolo-2′-deoxycytidine (Table 2, ON1) or phenyl derivative of Py-dC (Table 2, ON 4) within TBA loop resulted in significant loss of inhibitory properties of analysed variants in comparison to parental compound. Interestingly, the AE decreases with the increase in aromatic character of the T7 modification. Although the TGT loop is generally said not to be directly involved in binding to thrombin exosite I, one of the models assumes that simultaneous interactions with more than one thrombin molecules are possible [7,38,39,40]. In this arrangement, the TGT loop is bound to exosite II via electrostatic interactions between TBA phosphates and basic residues of the thrombin. What is more, it was also observed that binding of ligands to each exosite could provoke changes in the environment of the catalytic site and influence thrombin activity [41]. On the other hand, the change of binding mode of the TBA modified with 5-nitroindole residues could also be possible and was previously reported by the Timofeev group [42]. Taking the above into account, it could be assumed that the specific character of ON3 substitution could result in more efficient inhibition of the thrombin via improved interactions with one of its exosites. Furthermore, in 2012, Borbone et al. reported favorable effects of acyclic thymidine mimicked with a hydrophobic five-membered ring fused on thymidine at the T7 position [32]. The TBA variant appeared to be a more efficient inhibitor of thrombin activity in comparison to the unmodified aptamer. Molecular modeling presented by the same scientific group showed that different binding modes of the modified TBA T7 variant are possible and are not a well-understood allosteric mechanism of thrombin action, including long-range interactions between exosites that can be activated, influencing biological activity of the modified TBA. The presence of a flexible bicyclic thymidine derivative might facilitate exposure of the phosphate backbone towards the solvent, allowing ionic interactions with protein. Additionally, the molecular interactions between the modified residue with the hydrophobic cleft of exosite I might be improved. Surprisingly, the modified TBA variants presented herein, despite increasing aromatic and hydrophobic character of T7 residue, have negative effects on the prolongation of thrombin time in reference to the unmodified TBA. The only exception is decyl Py-dC modified TBA, which appeared to have improved anticoagulant properties in comparison to parental TBA. Different behavior of the Py-dC modified TBA variants can be due to decreased flexibility of the sugar residue and a more aromatic character of Py-dC derivatives in reference to acyclic thymidine mimics described by Scuotto group, and consequently by a worse ability to adjust the TGT loop to orientation, which is more optimal for TBA–thrombin interactions. However, observed beneficial influence of the presence of decyl aliphatic side-chain (ON3) on TBA anticoagulant properties might be justified by the relatively large length of the substituent. Nevertheless, the origin of improved activity of ON3 in comparison to TBA is not fully understood and requires further, comprehensive investigations.

### 2.4. Antiproliferative Properties of Modified Thrombin Binding Aptamer Variants

Recently, the attention of many research groups has focused on the antiproliferative potential of guanosine-rich oligonucleotides. These molecules are usually G-quadruplexes that are able to interact specifically with proteins involved in the cell cycle, which could restrain cell proliferation [43,44,45]. What is more, it has also been demonstrated that some modified TBA variants with decreased anticoagulant properties are characterized by favorable antiproliferative activity [21,31].

**Figure 3:**
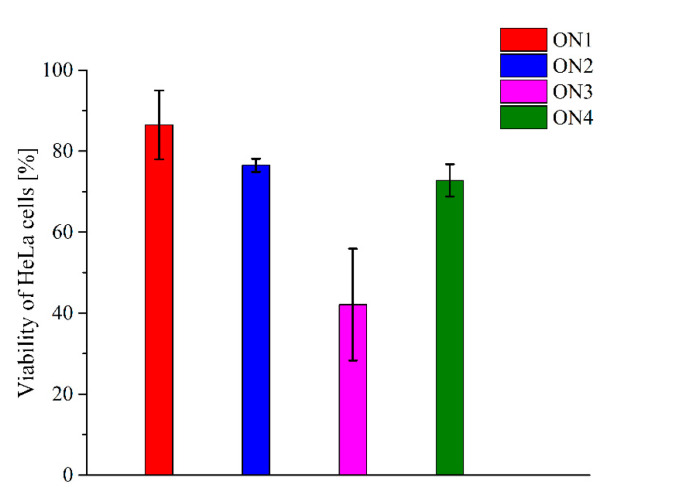
The antiproliferative activity of G-quadruplexes. The viability of HeLa cells in the presence of TBA variants modified with bicyclic Py-dC (ON1, red bar), octyl Py-dC (ON2, blue bar), decyl Py-dC (ON3, magenta bar) and phenyl Py-dC (ON4, green bar). The presented results are the mean values with ± SEM from two independent experiments.

Based on the above reports, determination of the abilities of modified TBA variants to inhibit cervical cancer cell line (HeLa) growth was performed. In order to realize this aim, the real-time cellular impedance assay was applied. The approach enables the assessment of cell proliferation based on the impedance of the electron flow caused by the presence of adherent cells on the surface of electrodes localized on the bottom of the cell culture plates. The degree of impedance is proportional to the percentage of viable cells in the experimental plates. The analysis was carried out for the aptamers at a 10 mM concentration. The results of the experiment indicate that the most efficient HeLa cell growth inhibitor was the TBA variant modified with a decyl derivative of Py-dC containing a long aliphatic, linear side-chain (Figure 3, ON3, HeLa cell viability was around 42%). The reduction in HeLa cell proliferation was also observed for ON2 and ON3, which possess octyl and phenyl derivative of Py-dC, respectively. However, the effect was moderate and the cell survivability was around 76% and 73%, respectively. Relatively low limitation of cell proliferation has been reported for the TBA variant with a bicyclic Py-dC substitution (ON1). As a consequence, almost 87% of HeLa cells were able to pass through in the presence of the ON1 aptamer.

## 3. Materials and Methods

### 3.1. Chemical Synthesis of Oligonucleotides

The synthesis of all oligonucleotides was performed on an automated RNA/DNA synthesizer (MerMade 12, BioAutomation, LGC Biosearch Technologies, Kenning Ct Plano, USA) with the application of standard phosphoramidite chemistry and commercially available nucleoside phosphoramidites GenePharma Co., Ltd, Suzhou, China) [46]. Further oligonucleotide preparation, i.e., deprotection and purification of unmodified TBA or its modified variants containing nucleotide derivative with pyrrolo-2’-deoxycytidine residues functionalized with various side-chains, was conducted in accordance with previously published protocols [16,29,47]. The 12% denaturing polyacrylamide (Bio-Rad, Hercules, CA, USA) gel was used to determine the purity of oligonucleotides, which was assessed to be above 95%. What is more, the matrix-assisted laser desorption ionization–time-of-flight mass spectrometry (MALDI TOF, Bruker Autoflex, Billerica, MA, USA) was applied to confirm the composition of all oligonucleotides.

### 3.2. UV Melting Analysis

The nine different concentrations of oligonucleotide solutions in the range of 10^−5^ −10^−7^ M were prepared in a buffer containing 100 mM potassium chloride (BioShop, Burlington, Kanada), 20 mM sodium cacodylate (BioShop, Burlington, Kanada), and 0.5 mM Na_2_EDTA (BioShop, Burlington, Kanada), with pH 7.0. The oligonucleotide absorbance at 80 °C was used to estimate the concentrations of single-stranded oligonucleotides, and the OligoAnalyzer (IDT, Coralville, USA) was applied to determine their extinction coefficients [48]. The samples denaturation was performed at 95 °C for 3 min, followed by overnight cooling to room temperature. The JASCO V-650 (Cremella (LC) Italy) spectrophotometer equipped with a thermoprogrammer was applied to obtain absorbance versus temperature curves. The UV melting measurements were performed at 295 nm in the temperature range of 4−90 °C with a heating rate of 0.2 °C/min. The MeltWin 3.5 software (Copyright 1995, 1996, Jeffrey A. McDowell, USA) was used to analyze the melting curves and to determine the melting temperatures via the application of nonlinear curve fitting.

### 3.3. Circular Dichroism Spectra

The JASCO J-815 spectropolarimeter (Cremella (LC), Italy) was applied to record the CD spectra. The oligonucleotide samples were prepared in 3.2 μM concentration in a buffer containing 100 mM potassium chloride (BioShop, Burlington, Kanada), 20 mM sodium cacodylate (BioShop, Burlington, Kanada), and 0.5 mM Na_2_EDTA (BioShop, Burlington, Kanada), pH 7.0. The samples denaturation was conducted at 95 °C for 3 min, followed by gradual, overnight cooling to room temperature. The measurements were performed at 4 °C and 37 °C in the 205−320 nm wavelength range, in triplicate. The resultant spectrum was obtained via subtraction of buffer spectra from the sample spectra. The Origin 8.0 software (OriginLab Corporation, Northampton, USA) was used to analyze obtained data.

### 3.4. Thermal Difference Spectra

The JASCO V-650 spectrophotometer (Cremella (LC) Italy) equipped with a thermoprogrammer was used to perform TDS measurements. The 28 µM concentration of each sample was prepared in a buffer containing 100 mM potassium chloride (BioShop, Burlington, Kanada), 20 mM sodium cacodylate (BioShop, Burlington, Kanada), and 0.5 mM Na_2_EDTA (BioShop, Burlington, Kanada) (pH 7.0). The measurements were performed at 4 and 90 °C in the 220 to 335 nm wavelength range, in triplicate. The spectra were recorded with 1000 nm/min scan speed and 1 nm data interval. The Origin 8.0 software (OriginLab Corporation, Northampton, USA) was used to obtain the final spectra via subtraction of the low-temperature absorbance spectra from the high-temperature absorbance spectra. The spectra normalization was performed by dividing the data by its maximum value [36].

### 3.5. Thrombin Time Assay

The measurements of anticoagulant properties of analyzed TBA variants was conducted using a coagulometer K-3002 Optic (Kselmed, Grudziądz, Poland) and commercially available Dia-TT kit (DIAGON, Budapest, Hungary). The 0.33 μM concentration of each oligonucleotide was prepared in 100 μL of Dia-TT reagent. The resultant samples were incubated at 37 °C for 5 min and after that positioned at a sample well of the coagulometer. Next, 100 μL of blood reagent Dia-CONT I (DIAGON, Budapest, Hungary) was added. The anticoagulant effect was calculated by subtraction of the thrombin time of plasma with aptamers from the thrombin time of control plasma. Thrombin time measurements for all oligonucleotides were performed in double replications.

### 3.6. The Real-Time Cellular Impedance Assay

The potential of TBA variants to restrain cell growth was determined via the real-time cellular impedance assay. The 100 mM oligonucleotide solutions were prepared in 1x PBS buffer with 100 mM KCl. The samples denaturation was conducted at 95 °C for 3 min, followed by overnight cooling to room temperature. The cervical carcinoma cells (HeLa cell line, from ATCC (American Type Culture Collection)), seeded in an electronic 16-well plate in seeding concentration 1000 cells/well in 200 mL of RPMI 1640 medium (Sigma-Aldrich, Saint Louis, MO, USA) with 10% fetal bovine serum (Sigma-Aldrich, Saint Louis, MO, USA) and 1x vitamin solution (Sigma-Aldrich, Saint Louis, MO, USA) were used to perform the measurements. The plates were incubated in the Agilent xCELLigence Real-Time Cell Analysis (RTCA) MP instrument at 37 °C, 5% CO_2_, and a relative humidity of 100% for 24 h. Next, the 10mM concentration of the chosen TBA variant (final working volume: 220 mL) was added to HeLa cells and 7-day incubation in the Agilent xCELLigence Real-Time Cell Analysis (RTCA) MP instrument (Agilent, Santa Clara, MO, USA) with constant growth monitoring was performed. All the appropriate control reactions were conducted. The RTCA software Pro (Agilent, Santa Clara, MO, USA) and Microsoft Excel 2013 software (Microsoft, Albuquerque, NM, USA) were applied in data analysis. The resultant data with ± SD constitute the mean values from two independent measurements.

## 4. Conclusions

In this paper, the incorporation of pyrrolo-2’-deoxycytidine (Py-dC) and its derivatives into a TBA TGT loop, along with comprehensive physicochemical and biological studies, have been presented for the first time.

The most favorable influence on anticoagulant properties of the TBA had the substitution of T7 with a decyl derivative of Py-dC containing a long aliphatic, linear side-chain. Importantly, the inhibitory effect of this aptamer was improved in comparison to the parental TBA compound. What is more, the same TBA variant also exhibited the potential to inhibit HeLa cell line growth. The presence of pyrrolo-2’-deoxycytidine (Py-dC) and its derivatives caused minor perturbation in the G-quadruplex thermodynamic properties without affecting the G-quadruplex folding topology. The structural analysis revealed that all modified TBA variants possessed an antiparallel G-quadruplex structure at physiological temperature.

The results described herein confirm that introduction of bicyclic 2′-deoxycytidine derivatives could be a suitable approach to modulate TBA biological properties with preservation of its structure. What is more, the data might greatly contribute to the broadening of general knowledge about modified G-quadruplexes and their structure–activity relationship but also to the development of novel TBA derivatives with superior anticoagulant or anticancer potential.

## Figures and Tables

**Table 1:** Thermodynamic parameters of TBA and TBA variants.

Name	Sequence (5ʹ-3ʹ)	Average of Curve Fits ^a^					
		−ΔH° (kcal/mol)	−ΔS° (eu)	ΔG°_37_ (kcal/mol)	T_M_ (°C)	ΔΔG°_37_ (kcal/mol)	ΔT_M_ (°C)
TBA	GGTTGGTGTGGTTGG	41.2 ± 0.9	127.2 ± 2.7	−1.74 ± 0.02	50.7	0	0
ON1	GGTTGG**1**GTGGTTGG	37.2 ± 0.5	115.3 ± 1.5	−1.49 ± 0.03	50.0	0.25	−0.7
ON2	GGTTGG**2**GTGGTTGG	38.6 ± 1.5	119.5 ± 4.8	−1.57 ± 0.05	50.1	0.17	−0.6
ON3	GGTTGG**3**GTGGTTGG	39.6 ± 1.5	122.7 ± 4.7	−1.51 ± 0.03	49.3	0.23	−1.4
ON4	GGTTGG**4**GTGGTTGG	29.6 ± 1.1	91.7 ± 3.6	−1.17 ± 0.02	49.8	0.57	−0.9

^a^ buffer: 100 mM KCl, 20 mM sodium cacodylate, 0.5 mM EDTA(Na)_2_, pH 7.0.

**Table 2:** Anticoagulant properties of TBA and its variants.

Oligomer	Sequence	AE ^a^ (s)
TBA	GGTTGGTGTGGTTGG	26.2
ON1	GGTTGG**1**GTGGTTGG	8.0
ON2	GGTTGG**2**GTGGTTGG	20.0
ON3	GGTTGG**3**GTGGTTGG	30.0
ON4	GGTTGG**4**GTGGTTGG	9.4

^a^ counted by subtraction of TT value for control plasma without aptamers from TT value for plasma with aptamers.

## Data Availability

All data are presented through the manuscript; no databases were utilized.

## References

[1] Santosh B., Yadava P.K. (2014). Nucleic Acid Aptamers: Research Tools in Disease Diagnostics and Therapeutics. BioMed Res. Int..

[2] Kotkowiak W., Pasternak A. (2021). Beyond G-Quadruplexes—The Effect of Junction with Additional Structural Motifs on Aptamers Properties. Int. J. Mol. Sci..

[3] Zhang N., Chen Z., Liu D., Jiang H., Zhang Z.-K., Lu A., Zhang B.-T., Yu Y., Zhang G. (2021). Structural Biology for the Molecular Insight between Aptamers and Target Proteins. Int. J. Mol. Sci..

[4] Gelinas A.D., Davies D.R., Janjic N. (2016). Embracing Proteins: Structural Themes in Aptamer-Protein Complexes. Curr. Opin. Struct. Biol..

[5] Bock L.C., Griffin L.C., Latham J.A., Vermaas E.H., Toole J.J. (1992). Selection of Single-Stranded DNA Molecules That Bind and Inhibit Human Thrombin. Nature.

[6] Wang K.Y., Krawczyk S.H., Bischofberger N., Swaminathan S., Bolton P.H. (1993). The Tertiary Structure of a DNA Aptamer Which Binds to and Inhibits Thrombin Determines Activity. Biochemistry.

[7] Padmanabhan K., Tulinsky A. (1996). An Ambiguous Structure of a DNA 15-Mer Thrombin Complex. Acta Crystallogr..

[8] Posma J.J.N., Posthuma J.J., Spronk H.M.H. (2016). Coagulation and Non-Coagulation Effects of Thrombin. J. Thromb. Haemost..

[9] Adams G.N., Rosenfeldt L., Frederick M., Miller W., Waltz D., Kombrinck K., McElhinney K.E., Flick M.J., Monia B.P., Revenko A.S. (2015). Colon Cancer Growth and Dissemination Relies upon Thrombin, Stromal PAR-1, and Fibrinogen. Cancer Res..

[10] Zhou S., Xiao W., Pan X., Zhu M., Yang Z., Zhang F., Zheng C. (2014). Thrombin Promotes Proliferation of Human Lung Fibroblasts via Protease Activated Receptor-1-Dependent and NF-ΚB-Independent Pathways. Cell Biol. Int..

[11] Coppens M., Eikelboom J.W., Gustafsson D., Weitz J.I., Hirsh J. (2012). Translational Success Stories: Development of Direct Thrombin Inhibitors. Circ. Res..

[12] Crawley J.T.B., Zanardelli S., Chion C.K.N.K., Lane D.A. (2007). The Central Role of Thrombin in Hemostasis. J. Thromb. Haemost..

[13] Russo Krauss I., Merlino A., Giancola C., Randazzo A., Mazzarella L., Sica F. (2011). Thrombin-Aptamer Recognition: A Revealed Ambiguity. Nucleic Acids Res..

[14] Pica A., Russo Krauss I., Merlino A., Nagatoishi S., Sugimoto N., Sica F. (2013). Dissecting the Contribution of Thrombin Exosite I in the Recognition of Thrombin Binding Aptamer. FEBS J..

[15] Mendelboum Raviv S., Horváth A., Aradi J., Bagoly Z., Fazakas F., Batta Z., Muszbek L., Hársfalvi J. (2008). 4-Thio-Deoxyuridylate-Modified Thrombin Aptamer and Its Inhibitory Effect on Fibrin Clot Formation, Platelet Aggregation and Thrombus Growth on Subendothelial Matrix. J. Thromb. Haemost..

[16] Kotkowiak W., Czapik T., Pasternak A. (2018). Novel Isoguanine Derivative of Unlocked Nucleic Acid-Investigations of Thermodynamics and Biological Potential of Modified Thrombin Binding Aptamer. PLoS ONE.

[17] Nallagatla S.R., Heuberger B., Haque A., Switzer C. (2009). Combinatorial Synthesis of Thrombin-Binding Aptamers Containing Iso-Guanine. J. Comb. Chem..

[18] Bonifacio L., Church F.C., Jarstfer M.B. (2008). Effect of Locked-Nucleic Acid on a Biologically Active g-Quadruplex. A Structure-Activity Relationship of the Thrombin Aptamer. Int. J. Mol. Sci..

[19] Zaitseva M., Kaluzhny D., Shchyolkina A., Borisova O., Smirnov I., Pozmogova G. (2010). Conformation and Thermostability of Oligonucleotide d(GGTTGGTGTGGTTGG) Containing Thiophosphoryl Internucleotide Bonds at Different Positions. Biophys. Chem..

[20] Smirnov I., Shafer R.H. (2000). Effect of Loop Sequence and Size on DNA Aptamer Stability. Biochemistry.

[21] Scuotto M., Rivieccio E., Varone A., Corda D., Bucci M., Vellecco V., Cirino G., Virgilio A., Esposito V., Galeone A. (2015). Site Specific Replacements of a Single Loop Nucleoside with a Dibenzyl Linker May Switch the Activity of TBA from Anticoagulant to Antiproliferative. Nucleic Acids Res..

[22] Pecoraro A., Virgilio A., Esposito V., Galeone A., Russo G., Russo A. (2020). UL3 Mediated Nucleolar Stress Pathway as a New Mechanism of Action of Antiproliferative G-Quadruplex TBA Derivatives in Colon Cancer Cells. Biomolecules.

[23] Esposito V., Russo A., Vellecco V., Bucci M., Russo G., Mayol L., Virgilio A., Galeone A. (2018). Thrombin Binding Aptamer Analogues Containing Inversion of Polarity Sites Endowed with Antiproliferative and Anti-Motility Properties against Calu-6 Cells. Biochim. Biophys. Acta. Gen. Subj..

[24] Esposito V., Russo A., Amato T., Vellecco V., Bucci M., Mayol L., Russo G., Virgilio A., Galeone A. (2018). The “Janus Face” of the Thrombin Binding Aptamer: Investigating the Anticoagulant and Antiproliferative Properties through Straightforward Chemical Modifications. Bioorg. Chem..

[25] Virgilio A., Esposito V., Pecoraro A., Russo A., Vellecco V., Pepe A., Bucci M., Russo G., Galeone A. (2020). Structural Properties and Anticoagulant/Cytotoxic Activities of Heterochiral Enantiomeric Thrombin Binding Aptamer (TBA) Derivatives. Nucleic Acids Res..

[26] Riccardi C., Meyer A., Vasseur J.-J., Russo Krauss I., Paduano L., Morvan F., Montesarchio D. (2020). Fine-Tuning the Properties of the Thrombin Binding Aptamer through Cyclization: Effect of the 5’-3’ Connecting Linker on the Aptamer Stability and Anticoagulant Activity. Bioorg. Chem..

[27] Bao H.-L., Ishizuka T., Yamashita A., Furukoji E., Asada Y., Xu Y. (2021). Improving Thermodynamic Stability and Anticoagulant Activity of a Thrombin Binding Aptamer by Incorporation of 8-Trifluoromethyl-2’-Deoxyguanosine. J. Med. Chem..

[28] Berry D.A., Jung K.-Y., Wise D.S., Sercel A.D., Pearson W.H., Mackie H., Randolph J.B., Somers R.L. (2004). Pyrrolo-DC and Pyrrolo-C: Fluorescent Analogs of Cytidine and 2′-Deoxycytidine for the Study of Oligonucleotides. Tetrahedron Lett..

[29] Jahnz-Wechmann Z., Lisowiec-Wachnicka J., Framski G., Kosman J., Boryski J., Pasternak A. (2017). Thermodynamic, Structural and Fluorescent Characteristics of DNA Hairpins Containing Functionalized Pyrrolo-2’-Deoxycytidines. Bioorg. Chem..

[30] Pasternak A., Hernandez F.J., Rasmussen L.M., Vester B., Wengel J. (2011). Improved Thrombin Binding Aptamer by Incorporation of a Single Unlocked Nucleic Acid Monomer. Nucleic Acids Res..

[31] Kotkowiak W., Lisowiec-Wachnicka J., Grynda J., Kierzek R., Wengel J., Pasternak A. (2018). Thermodynamic, Anticoagulant, and Antiproliferative Properties of Thrombin Binding Aptamer Containing Novel UNA Derivative. Mol. Ther. Nucleic Acids.

[32] Borbone N., Bucci M., Oliviero G., Morelli E., Amato J., D’Atri V., D’Errico S., Vellecco V., Cirino G., Piccialli G. (2012). Investigating the Role of T7 and T12 Residues on the Biological Properties of Thrombin-Binding Aptamer: Enhancement of Anticoagulant Activity by a Single Nucleobase Modification. J. Med. Chem..

[33] Migliore M.D., Zonta N., McGuigan C., Henson G., Andrei G., Snoeck R., Balzarini J. (2007). Synthesis and Antiviral Activity of the Carbocyclic Analogue of the Highly Potent and Selective Anti-VZV Bicyclo Furano Pyrimidines. J. Med. Chem..

[34] Russo Krauss I., Merlino A., Randazzo A., Novellino E., Mazzarella L., Sica F. (2012). High-Resolution Structures of Two Complexes between Thrombin and Thrombin-Binding Aptamer Shed Light on the Role of Cations in the Aptamer Inhibitory Activity. Nucleic Acids Res..

[35] Giraldo R., Suzuki M., Chapman L., Rhodes D. (1994). Promotion of Parallel DNA Quadruplexes by a Yeast Telomere Binding Protein: A Circular Dichroism Study. Proc. Natl. Acad. Sci. USA.

[36] Mergny J.-L., Li J., Lacroix L., Amrane S., Chaires J.B. (2005). Thermal Difference Spectra: A Specific Signature for Nucleic Acid Structures. Nucleic Acids Res..

[37] Kotkowiak W., Wengel J., Scotton C.J., Pasternak A. (2019). Improved RE31 Analogues Containing Modified Nucleic Acid Monomers: Thermodynamic, Structural, and Biological Effects. J. Med. Chem..

[38] Nagatoishi S., Isono N., Tsumoto K., Sugimoto N. (2011). Loop Residues of Thrombin-Binding DNA Aptamer Impact G-Quadruplex Stability and Thrombin Binding. Biochimie.

[39] Pagano B., Martino L., Randazzo A., Giancola C. (2008). Stability and Binding Properties of a Modified Thrombin Binding Aptamer. Biophys. J..

[40] Cai B., Yang X., Sun L., Fan X., Li L., Jin H., Wu Y., Guan Z., Zhang L., Zhang L. (2014). Stability and Bioactivity of Thrombin Binding Aptamers Modified with D-/L-Isothymidine in the Loop Regions. Org. Biomol. Chem..

[41] Bock P.E., Panizzi P., Verhamme I.M.A. (2007). Exosites in the Substrate Specificity of Blood Coagulation Reactions. J. Thromb. Haemost..

[42] Tsvetkov V.B., Varizhuk A.M., Pozmogova G.E., Smirnov I.P., Kolganova N.A., Timofeev E.N. (2015). A Universal Base in a Specific Role: Tuning up a Thrombin Aptamer with 5-Nitroindole. Sci. Rep..

[43] Bates P.J., Laber D.A., Miller D.M., Thomas S.D., Trent J.O. (2009). Discovery and Development of the G-Rich Oligonucleotide AS1411 as a Novel Treatment for Cancer. Exp. Mol. Pathol..

[44] Choi E.W., Nayak L.V., Bates P.J. (2010). Cancer-Selective Antiproliferative Activity Is a General Property of Some G-Rich Oligodeoxynucleotides. Nucleic Acids Res..

[45] Roxo C., Kotkowiak W., Pasternak A. (2021). G4 Matters—the Influence of G-Quadruplex Structural Elements on the Antiproliferative Properties of G-Rich Oligonucleotides. Int. J. Mol. Sci..

[46] Roy S., Caruthers M. (2013). Synthesis of DNA/RNA and Their Analogs via Phosphoramidite and H-Phosphonate Chemistries. Molecules.

[47] Lopez-Gomollon S., Nicolas F.E. (2013). Purification of DNA Oligos by Denaturing Polyacrylamide Gel Electrophoresis (PAGE). Meth. Enzymol..

[48] Mergny J.-L., Lacroix L. (2009). UV Melting of G-Quadruplexes. Curr. Protoc. Nucleic Acid Chem..

